# Cranial Electrotherapy Stimulation to Improve the Physiology and Psychology Response, Response-Ability, and Sleep Efficiency in Athletes with Poor Sleep Quality

**DOI:** 10.3390/ijerph19041946

**Published:** 2022-02-09

**Authors:** Wen-Dien Chang, Yung-An Tsou, Yi-Ying Chen, Bao-Lien Hung

**Affiliations:** 1 Department of Sport Performance, National Taiwan University of Sport, Taichung 404401, Taiwan; c252522002@gmail.com; 2 Department of Otolaryngology-Head and Neck Surgery, China Medical University Hospital, Taichung 40402, Taiwan; d22052121@gmail.com; 3 Department of Audiology and Speech-Language Pathology, Asia University, Taichung 41354, Taiwan; 4 School of Medicine, China Medical University, Taichung 40402, Taiwan; 5 Department of Sports Medicine, China Medical University, Taichung 406040, Taiwan; blhoung@mail.cmu.edu.tw

**Keywords:** cranial electrotherapy stimulation, heart rate variability, sleep efficiency

## Abstract

Athletes often have poor sleep quality before a competition. Sleep quality can stabilize mood and improve sports performance. The randomized controlled study explored the effects of cranial electrotherapy stimulation (CES) on the physiology, psychology, response-ability, and sleep quality of athletes who had poor sleep quality before a competition. Athletes who had poor sleep quality (Pittsburgh Sleep Quality Scale score > 5) and had a competition in less than 2 months were recruited. The athletes were grouped into the CES group, which received a 2-week CES treatment (*n* = 20, age = 21.55 ± 2.26 years), and a placebo group (*n* = 20, age = 21.05 ± 1.46 years), which received a 2-week sham CES treatment. We performed biochemical analysis, a simple reaction time test, choice reaction time tests, the Profile of Mood States, heart rate variability (HRV), and an Actigraphy activity recorder to measure outcomes before and after the interventions. Our results revealed no significant differences in blood urea nitrogen, creatine phosphate, testosterone, cortisol, and saliva pH between and within groups (*p* > 0.05). Significant decreases in negative mood states (i.e., anger, tension, and depression) and choice reaction time in the CES group were noted (*p* < 0.05), moreover, the anger, tension, and depression mood decreased from 0.36 ± 0.45 (95% CI = 0.16–0.55), 1.62 ± 0.97 (95% CI = 1.19–2.04), and 1.67 ± 1.06 (95% CI = 1.20–2.13) to 0.11 ± 0.20 (95% CI = 0.02–0.19, *p* = 0.03), 1.12 ± 0.74 (95% CI = 0.79–1.44, *p* = 0.04), and 0.81 ± 0.75 (95% CI = 0.48–1.13, *p* = 0.001), respectively. Additionally, choice reaction time was decreased from 420.85 ± 41.22 ms (95% CI = 402.78–438.91) to 399.90 ± 36.71 ms (95% CI = 383.81–415.98, *p* = 0.04) and was also noted in the CES group. For HRV, and Actigraphy activity for sleep measure, the low-frequency (LF)/high-frequency (HF) ratios changed from 1.80 ± 1.39 (95% CI = 1.19–2.40) to 1.21 ± 0.73 (95% CI = 0.89–1.53, *p* = 0.10), and sleep efficiency decreased from 87.94 ± 6.76% (95% CI = 84.97–90.90) to 81.75 ± 9.62% (95% CI = 77.53–85.96, *p* = 0.02) in the CES group. The change in LF/HF after the trial were found between CES and placebo groups (p < 0.05). Yet, the decrease in sleep efficiency in the placebo group were noted (*p* < 0.05). However, we found that the regression line for sleep efficiency was decreased less during the study while using CES. The CES intervention could reduce negative emotions, improve choice reaction times, enhance the parasympathetic and sympathetic nerve activity imbalances, and slow sleep efficiency deterioration. Regardless, small effect sizes of the application of CES on psychology response, response-ability, and sleep efficiency were concluded in athletes with poor sleep quality before a competition.

## 1. Introduction

Sleep is a restorative mechanism that benefits athletes’ physiology and psychology [1]. In a survey of 283 elite Australian athletes, 64% were reported to have experienced at least one sleep problem before a major competition in the past year [2]. Many sports injuries are due to pre-competition nervousness. The effects of poor sleep on individual and team performance are different. Athletes often have trouble sleeping before competitions [2]. Precompetition, overtraining, or adaptability problems often cause athletes to be sleep-deprived or have poor sleep quality. Some studies found that increased training and match schedules during the international competition could affect the sleep quality and nocturnal heart rate variability (HRV) in female athletes [3,4]. Figueiredo et al. also noted the sleep pattern and HRV were changed in youth athletes during the competition, and their sleep duration and training loading had a negative relationship [5]. Leeder et al. used the Actigraph activity recorder to measure the sleep of 47 Olympians and compared them with a control group of 20 nonathletes. They found that elite athletes had worse sleep quality and difficulty falling asleep before a competition [6]. Costa et al. indicated that the change in Actigraph activity and HRV could provide adequate information about nocturnal sleep patterns and autonomic nervous activity, reflecting athletes’ psychophysiological recovery state [4]. Upcoming competitions precipitate lack of sleep or sleep deprivation in athletes [7]. Anxiety before a competition and overtraining resulted in poor sleep quality [8]. In this condition, physical and psychological recovery management is required to reduce the risk of poor sleep quality due to overtraining or excessive fatigue and decrease the risk of sports injury [9]. Dinges et al. assessed sleep loss in 16 healthy young adults. Their sleep time was reduced by 33% for seven consecutive nights for an average of 4.98 h per night [6]. The results showed that loss of sleep time caused cumulative and increasing daytime sleepiness, fatigue, and negative emotions [10]. Jarraya et al. indicated that partial sleep deprivation might decrease the level of vigilance, including decreases in the performances of concentration and motion reaction time [11]. Blumert et al. indicated that negative moods such as confusion and fatigue were increased, and that vitality was decreased in weightlifters after 24 h of sleep deprivation [12]. Therefore, sleep loss can cause physiological and psychological problems and thus affect athletes’ sports performance.

Cranial electrotherapy stimulation (CES) is a non-invasive neuromodulation technique that can manage sleep problems—it is an electrosleep therapy [13]. CES uses a feeble current (<1 mA) to influence brain excitation [13]. A weak current passing through the brain changes cell membrane potentials and the neuron excitability threshold [14]. CES could stimulate specific neurotransmitters and hormones in the brain related to anxiety, depression, and insomnia [15]. A meta-analysis revealed that CES had moderating effects on insomnia (effect size = 0.64) and that the improvement rate for insomnia was 50–93% [16]. Elite athletes had a higher risk of poor sleep quality because of psychological stresses such as anxiety before competitions [2]. A study indicated that 65.8% of athletes had insomnia before major competitions [17]. Athletes sometimes use medications to improve rest and accelerate recovery when experiencing poor sleep quality [18]. This misuse of drugs often qualifies as doping in sports. Therefore, physiotherapy, such as CES might be a safe and effective alternative for athletes attempting to manage sleep problems before a competition. However, studies on the efficacy of CES for athletes with sleep problems are rare. This study aimed to explore the effects of CES on athletes with poor sleep quality before a competition by measuring changes in sleep quality physiological and psychological responses. We hypothesized that CES would enhance the athletes’ sleep quality and psychophysiological effects before the competition. Additionally, we expected the change in sleep efficiency during the study and the associations of the related variables.

## 2. Materials and Methods

The Institutional Review Board of China Medical University and Hospital (CMUH) approved this randomized controlled study. Informed consent was obtained from the participants. Athletes, who were Taiwanese, were recruited before competitions and screened using the Pittsburgh Sleep Quality Index (PSQI), polysomnography (PSG), and the Epworth sleepiness scale (ESS). The PSQI is a self-rated questionnaire containing seven items: subjective sleep quality, sleep latency, sleep duration, habitual sleep efficiency, sleep disturbances, use of sleep medication, and daytime dysfunction. It has high internal consistency and reliability (Cronbach’s α = 0.83) and good test–retest reliability (correlation coefficient r = 0.85) for assessing sleep quality and disturbances [19]. The ESS is an eight-item questionnaire assessing the propensity to fall asleep during the day and screen for poor sleep quality [20]. Because the participants were Taiwanese, the Chinese version of PSQI (reliability coefficient, r = 0.82–0.83) and ESS (test–retest reliability, r = 0.67) were used to assess for sleep problems [19,21], and both questionnaires had high internal consistency and reliability. The PSQI and ESS were used to screen the participants by one physician. PSG is used to assess the current study’s abnormal sleep pattern and is considered an accurate assessment method for various sleep disorders [22]. All patients were required to sleep in the CMUH sleep medicine center for one night. Physiological data collected during sleep included electroencephalograms, electrocardiograms, oxygen saturation, airflow signals, respiration, and sleep patterns. The same otolaryngologist assessed the data.

### 2.1. Participants

Healthy athletes who had a competition in less than 2 months and had poor sleep quality (total PSQI ≥ 5 and ESS ≥ 10), which is defined as the study of Swinbourne et al. [23], were eligible for inclusion in the study. The athletes are recruited from sports teams in a sports university, including taekwondo, boxing, discus throwing, wrestling, cycling, track and field. The exclusion criteria were athletes with abnormal PSG findings such as sleep apnea (Apnea-Hypopnea Index, AHI > 10 times/h), narcolepsy, hypersomnia, or periodic limb movement syndrome [22], and athletes who were unable to complete the experimental procedure. The participants were randomly divided into CES and placebo groups and were randomly divided into two groups at a 1:1 ratio using a simple random sampling. The group allocation was concealed sequentially on the numbered cards and chosen by one independent researcher. On the reference of sample size reported by Feighner et al. [24], 19 participants were in each group. We estimated the sample size using the G*Power software (version 3.1.9.2; Heinrich-Heine-Universität, Düsseldorf, Germany). The effect size f of 0.25, the statistical power of 80%, and the α level of 0.05 were used to calculate, and a sample size of 32 (16 participants per group) was required [25]. Hence, the estimated sample size was set to at least 38 participants (19 participants per group) in the current study.

### 2.2. Study Procedure

**Figure 1:**
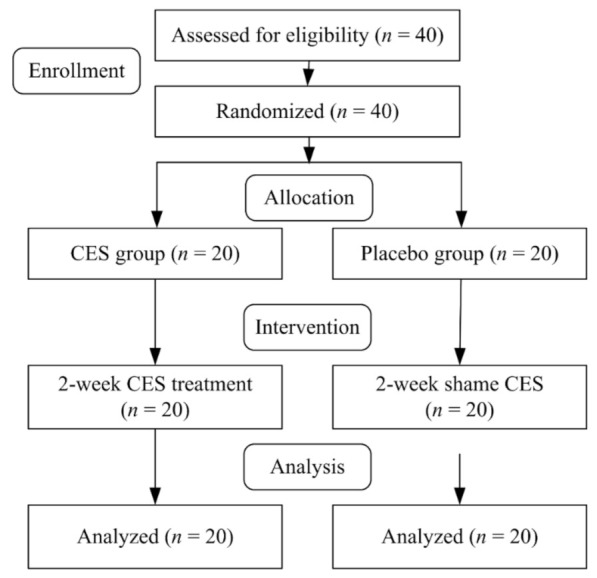
Study flow diagram.

The study procedure is diagrammed in Figure 1. Biochemical analysis, simple and choice reaction time, the Profile of Mood States (POMS), HRV, and an Actigraph activity recorder were used to measure relevant indicators before and after the intervention. Actigraph activity recorder was continually measured and recorded day and night for 14 days. The same researcher performed all assessments and interventions, and the same analyst analyzed the assessed data. All researchers were blinded to the allocation and intervention of participants.

### 2.3. Interventions

A CES device (Alpha-Stim, Electromedical Products International, Inc., TX, USA) was used to electrically stimulate the brain with an electrical current including bipolar and asymmetric waves (0.5 Hz, 100 μA, 50% duty cycle). These electrotherapy parameters were referred by the study of Kirsch et al. [26], reporting a clinical improvement of 65.3% for insomnia after using CES. The participants in the CES group were asked to place electrodes on their ear lobes and treat themselves for 60 min per day for 2 weeks. The placebo CES device was identical to the real CES device, but an electrical current did not output from the electrodes. The CES devices were prepared for the participants by one researcher. The device settings could not be changed during the study. Every day, a researcher interviewed the participants by telephone to monitor device usage and check for any adverse events.

### 2.4. Assessments

#### 2.4.1. Biochemistry Analysis

The participants’ saliva, blood, and urine were collected, and muscle fatigue and recovery biomarkers were assessed. Ten milliliters of blood were drawn from either the participants’ left or right median cubital vein. The blood was centrifuged at 3000 rpm for 10 min, and the upper layer of serum was stored in a −80 °C freezer [27]. After blood was drawn, urine and saliva were collected from the participants. Participants were asked to chew a sterile rubber to stimulate the salivary flow, and saliva was collected during 5-minute chewing [28]. At least, the urine was collected. All biochemistry samples were collected from 8 to 11 am and were analyzed in the hospital laboratory. Blood urea nitrogen, creatine phosphate, testosterone, cortisol levels, and saliva pH were measured before and after the intervention.

#### 2.4.2. Simple and Choice Reaction Time

Reaction time is an indicator of an athlete’s sports performance. The simple and choice reaction time tests were performed using the PsyToolkit online software [29]. The participants pressed a key in response to visual stimuli, and reaction times for simple and choice reaction tests were measured. The simple reaction time is measured when one visual symbol stimulus requires the response by pressing the computer keyboard. When there are correct and wrong visual symbol stimuli, the choice reaction time requires correct choice response by pressing the computer keyboard button [30].

#### 2.4.3. Profile of Mood State

The Chinese version of POMS is a questionnaire used to assess subjective mood states, and the translation items of POMS were clear and easily understood for Taiwanese [31]. The 30-item short-form POMS was used for assessment in this study. POMS measures perceived confusion, fatigue, anger, tension, depression, vigor, and self-esteem [32]. Participants were asked to rate their perceived mood on a scale from 0 (not at all) to 4 (extremely) for each question. These ratings were used to derive five subscales and total scores. The POMS questionnaire has high reliability (Cronbach’s α = 0.75–0.95) for self-assessment of mood [32].

#### 2.4.4. Heart Rate Variability

HRV was measured using an HRV monitor (Check-My-Heart, Daily Care BioMedical, Taoyuan, Taiwan). The adhesive electrodes (Kendall, MA, USA) were attached to both wrists, and HRV signals were recorded for 5 min. The recorded data were analyzed using HRV software (HRV analysis software, Daily Care BioMedical, Taoyuan, Taiwan). The resulting measure data could assess variations in autonomic nervous system activity [33]. In the frequency domain for signal processing, the high-frequency (HF) range was set to 0.15–0.4, and the low-frequency (LF) range was set to 0.04–0.15. Normalized LF and HF were considered to represent sympathetic and parasympathetic nerve activity, respectively. Furthermore, the LF/HF ratio represented the balance of parasympathetic and sympathetic nerve activities [34]. The standard deviation of normal-to-normal interval (SDNN) was also computed in the time domain [34].

#### 2.4.5. Actigraph Activity Measurement

Daily sleep condition and sleep efficiency over the 2-week trial were continually measured by wristwatch Actigraphy recorders (Actigraph GT3X, Pensacola, FL, USA), recorded at a sample rate of 60 Hz with epochs of 60 s [35]. The height and weight of each athlete were set in the wristwatch, which was worn on their non-dominant wrist at night. The wristwatch of Actigraph is a valid alternative method of sleep monitoring for elite athletes [36]. Degroote et al. provided some evidence of the validity of Actigraph GT3X to measure sleep duration [35]. A researcher verbally instructed the participants on using the device and collected sleep onset and end of sleep data for the 2 weeks in phone interviews. At the end of the 2 weeks, sleep data were retrieved and analyzed using ActiLife software (Actigraph, LLC, Fort Walton Beach, FL, USA). The sleep parameters (i.e., sleep latency, sleep efficiency, total minutes in bed, total sleep time, wake after sleep onset, number of awakenings, average awakening length, movement index, fragmentation index, and sleep fragmentation index) were analyzed using the Sadeh algorithm by the same researcher for all participants. The Sadeh algorithm analyzes sleep-related data using software-validated sleep quality in young adults [37].

### 2.5. Statistical Analysis

SPSS (version 25; SPSS Inc., Chicago, IL, USA) was used to analyze all data. Descriptive statistics were used to analyze demographic data, and all assessed variables were represented as means ± standard deviations. Differences in demographic and baseline data of the CES and placebo groups were compared using independent t-tests for continuous variables. Comparative analyses of the assessed variables of biochemistry values, POMS score, HRV, reaction time, and sleep analysis before and after the intervention were conducted using two-way analysis of covariance (ANCOVA) followed by the Bonferroni post hoc test. The within-subjects factor was the time, with two levels (pretrial and posttrial), and the between-subjects factor was the group, with two levels (CES and placebo groups). The time analysis revealed changes in pretrial and posttrial outcome measures. Effect size (ES) was calculated by using partial eta squared (ηp^2^) and classed a small (0.01), medium (0.06), and large (0.14) based on the rules of Cohen et al. [38] Changes in sleep efficiency during the trial in both groups were assessed using linear regression. The differences in proteinuria and urobilinogen were compared using a chi-square analysis. The changes on the related variables were used Pearson correlation coefficient for within-subjects correlations, which were classed as almost perfect (r ≥ 0.9), very large (r = 0.7–0.9), large (r = 0.5–0.7), moderate (r = 0.3–0.5) and small (r = 0.1–0.3) [39]. The significance level was set to *p* < 0.05 for all tests.

## 3. Results

In this study, 40 athletes completed the experimental trial, and no participants reported adverse reactions and dropped out. The athletes were recruited to participate from the college sports terms. As displayed in Table 1, the participants were assigned to the CES group (*n* = 20) or the placebo group (*n* = 20). Among the 40 athletes eligible for the study, insomnia occurred 1.5–4 times per week, and their total PSQI score was 7–10. Each item in PSQI were mean scores of 0.90 in subjective sleep quality, 1.88 in sleep latency, 1.21 in sleep duration, 1.71 in habitual sleep efficiency, 1.85 in sleep disturbances, 0 in use of sleep medication, and 1.52 in daytime dysfunction.

All participants completed the study trial with no dropouts or adverse events reported. Differences in demographic and baseline variables between the CES and placebo groups were evaluated. Table 2 demonstrates that no significant differences were observed in the demographic data between the groups (*p* > 0.05). Differences in total PSQI score, ESS, sleep architecture, periodic limb movement, lowest SpO_2_ (%), AHI, sleep onset latency, and sleep efficiency from PSG analysis were also not statistically significant (*p* > 0.05).

**Figure 2:**
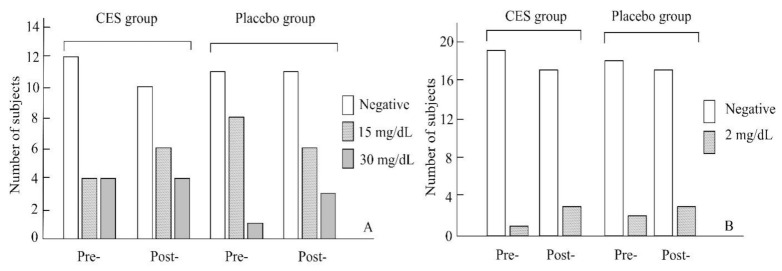
Changes in proteinuria (**A**) and urobilinogen (**B**) in the two groups.

Table 2 reveals no significant differences between the groups before or after the trial in blood urea nitrogen, creatine phosphate, testosterone, cortisol levels, or saliva pH (*p* > 0.05). Abnormal proteinuria was observed in 40% of the athletes in the CES group 45% of those in the placebo group pretrial and 50% of the athletes in the CES group, and 45% of those in the placebo group posttrial (Figure 2A). No statistical difference was observed for abnormal proteinuria before and after the trial (*p* > 0.05). Abnormal urobilinogen levels were observed for 5% of the athletes in the CES group and 15% of those in the placebo group pretrial and 10% of the athletes in the CES group, and 15% of those in the placebo group posttrial (Figure 2B). No statistical difference in pretrial and posttrial abnormal urobilinogen levels was observed (*p* > 0.05).

Changes in pretrial and posttrial POMS scores were found in the two groups (Table 3). Effects were found in total mood disturbance over time (F = 9.14, η*p*^2^ = 0.31, *p* = 0.007), between groups (F = 3.56, η*p*^2^ = 0.15, *p* = 0.07), and for time × group (F = 0.74, η*p*^2^ = 0.03, 95% CI = 86.22–98.63, *p* = 0.39). There was a significant main effect on total mood disturbance of time (*p* < 0.05). The post hoc tests revealed no significant differences in either group for total mood disturbance (*p* > 0.05). The main effects on anger, tension, and depression in POMS scores for time (F = 3.84, η*p*^2^ = 0.16, *p* = 0.05; F= 18.96, η*p*^2^ = 0.48, *p* = 0.001; F = 25.43, η*p*^2^ = 0.56, *p* = 0.001), group (F = 0.01, η*p*^2^ = 0.001, *p* = 0.93; F = 0.12, η*p*^2^ = 0.006, *p* = 0.73; F = 0.01, η*p*^2^ = 0.001, *p* = 0.93), and time × group (F = 5.80, η*p*^2^ = 0.23, 95% CI = 0.11–0.36, *p* = 0.02; F = 0.24, η*p*^2^ = 0.01, 95% CI = 1.10–1.72, *p* = 0.62; F = 5.56, η*p*^2^ = 0.21, 95% CI = 0.88–1.61, *p* = 0.02) were noted. The post hoc tests revealed significant differences for anger (*p* = 0.03), tension (*p* = 0.04) and depression (*p* = 0.001) in the pretrial and posttrial POMS scores in the CES group.

In the HRV analysis (Table 3), no significant differences were observed between the groups over time for heart rate or SDNN (*p* > 0.05). We found effects on LF, HF, and LF/HF for time (F = 0.12, η*p*^2^ = 0.04, *p* = 0.35; F =0.12, η*p*^2^ = 0.006, *p* = 0.73; F = 0.61, η*p*^2^ = 0.02, *p* = 0.44, respectively), group (F = 0.91, η*p*^2^ = 0.44, *p* = 0.35; F = 0.94, η*p*^2^ = 0.04, *p* = 0.34; F = 1.11, η*p*^2^ = 0.05, *p* = 0.31, respectively), and time × group (F = 4.03, η*p*^2^ = 0.16, 95% CI = 51.49–58.78, *p* = 0.05; F = 3.90, η*p*^2^ = 0.16, 95% CI = 41.15–48.44, *p* = 0.06; F = 1.82, η*p*^2^ = 0.08, 95% CI = 1.36–1.95, *p* = 0.19, respectively). In the CES group, LF decreased by 7.74 ± 20.02%, HF increased by 7.74 ± 20.02%, and LF/HF decreased from 1.81 ± 1.39 to 1.21 ± 0.73. In the placebo group, LF increased by 5.61 ± 14.31%, HF decreased by 5.43 ± 14.29%, and LF/HF increased from 1.76 ± 1.87 to 1.85 ± 1.15. The post hoc tests indicated a significant change in LF/HF after the trial between CES and placebo groups (*p* < 0.05).

Changes in pretrial and posttrial reaction time test scores for both groups are listed in Table 3. For simple reaction time, the main effects for time (F = 1.02, η*p*^2^ = 0.04, *p* = 0.32), group (F = 0.05, η*p*^2^ = 0.003, *p* = 0.81), and time × group (F = 1.78, η*p*^2^ = 0.08, 95% CI = 300.36–321.77, *p* = 0.19) were calculated. The post hoc tests revealed no significant differences within groups in simple reaction time (*p* > 0.05). For choice reaction time, we calculated the main effects for time (F = 1.23, η*p*^2^ = 0.05, *p* = 0.28), group (F = 1.44, η*p*^2^ = 0.06, *p* = 0.24), and time × group (F = 4.88, η*p*^2^ = 0.20, 95% CI = 403.40–430.19, *p* = 0.03). The post hoc tests showed a significant decrease in choice reaction time for the CES group (*p* = 0.04), and the participants in the CES group had a greater posttrial decrease in reaction time than did the participants in the placebo group (*p* = 0.04).

**Figure 3:**
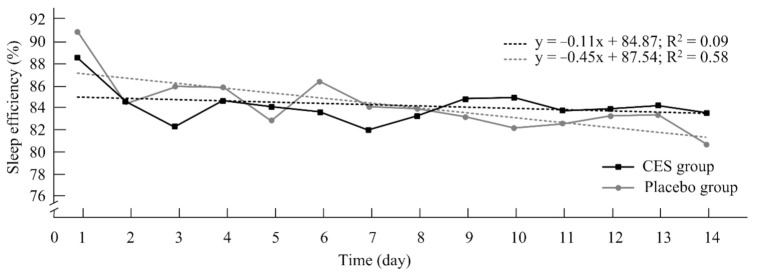
Changes in sleep efficiency slopes during the study.

The results of the sleep architecture analysis (Table 4) revealed the effects on total sleep time and number of awakenings for time (F = 22.01, η*p*^2^ = 0.52, *p* = 0.001; F = 24.87, η*p*^2^ = 0.55, *p* = 0.001, respectively), group (F = 0.31, η*p*^2^ = 0.02, *p* = 0.58; F = 0.01, η*p*^2^ = 0.001, *p* = 0.91, respectively), and time × group (F = 4.21, η*p*^2^ = 0.14, 95% CI =287.73–336.78, *p* = 0.04; F = 1.81, η*p*^2^ = 0.08, 95% CI = 14.71–19.94, *p* = 0.19, respectively). Sleep time and deep sleep time tended to decrease from before to after the trial. Effects were also observed for sleep efficiency over time (F = 27.76, η*p*^2^ = 0.58, *p* = 0.001), group (F = 0.10, η*p*^2^ = *p* = 0.76), and time × group (F = 0.21, η*p*^2^ =0.01, 95% CI = 82.16–88.15, *p* = 0.54), with a decrease in sleep quality observed from pretrial to posttrial. In addition, the post hoc tests revealed significant differences in sleep efficiency, total minutes in bed, total sleep time, and number of awakenings. Sleep quality was observed to decrease from before to after the trial in the placebo group (all *p* < 0.05), whereas changes in both sleep efficiency and number of awakenings were observed in the CES group (all *p* < 0.05). The slope of the regression line for sleep efficiency in the CES (R^2^ = 0.09) and placebo groups (R^2^ = 0.58) decreased over time, indicating that sleep quality decreased over the course of the study. However, the slope of the line decreased less for the CES group, indicating that sleep efficiency decreased less during the study (Figure 3). The difference in the sleep efficiency slope change was statistically significant (*p* = 0.02).

As our outcome findings, some variables, such as total mood disturbance, LF, HF, LF/HF, simple reaction time, choice reaction time, and sleep efficiency, had significant differences in CES and placebo groups. The within-subject correlations of these related variables in both groups are presented in Table 5. A large negative correlation was noted between the changes of total mood disturbance and sleep efficiency in the CES group (r = −0.51, *p* = 0.01). In CES and placebo groups, the change of LF had very large correction with the change of LF/HF (r = 0.95, *p* = 0.01; r = 0.89, *p* = 0.01, respectively). There were no significant correlations between the other variables (*p* > 0.05).

## 4. Discussion

CES is a physiotherapy agent and a non-invasive neuromodulation technique to improve sleep problems. Electrosleep therapy technologies use feeble currents of less than 1 mA to regulate the excitability of the brain [26]. CES can change cell membrane potentials and nerve excitement thresholds [14]. The use of low-intensity microcurrents to stimulate the brain may cause the brain to secrete neurotransmitters and hormones involved in anxiety, depression, and insomnia [40]. To the best of our knowledge, the current study is the first randomized controlled trial to investigate the use of CES for athletes with poor sleep quality pre-competition. We investigated the effects of CES by using biochemical analysis, simple and choice reaction time, the POMS, HRV, and Actigraphy to measure outcomes. Compared with the placebo group, the effect of CES use on anger and depression of POMS, LF/HF of HRV, choice reaction time, and some sleep parameters in Actigraphy had small effect sizes. The results reveal a significant decrease in anger, tension, and depression as measured by the POMS and a reduction in choice reaction time in the CES group (all *p* < 0.05). As the competition approached, the participants in the placebo group tend to have higher LF of HRV and decrease sleep efficiency. Although decreased sleep efficiency was also observed for participants in the CES group pre-competition, LF/HF of HRV was more stable to compare with placebo group.

The current study analyzed the changes in blood urea nitrogen, creatine phosphate, testosterone, cortisol levels, and saliva pH in both groups. The causes of the physiological changes could be due to sports training, competition intensity, or stress. However, a few studies have explored the relationship between sleep problems and physiological responses in athletes. The hormone cortisol is a physiological indicator of both stress and functional catabolism. Anderson et al. reported that the cortisol levels of athletes increase rapidly after exercise-related exhaustion before returning to normal values. Sustained elevated cortisol levels indicate that an athlete requires longer recovery times and that the athlete’s overall physiological function may be poor [41]. Blood urea nitrogen level, creatine phosphate level, and saliva pH were also reported to be indicators of physical fatigue in athletes [42]. No significant differences were observed for blood urea nitrogen, creatine phosphate, testosterone, cortisol levels, or saliva pH between or within study groups in the present study [42]. As the competition approached, only abnormal proteinuria and urobilinogen increased 10% and 5%, respectively. Elite athletes adapt to changes in sports training volume before competitions. Thus, these physical indicators were not observed to change significantly in the pre-competition period and did not influence sleep quality.

Competition causes physical and psychological stress in athletes due to pressure, overtraining, and fatigue recovery [43]. These stresses may decrease sports performance and cause overtraining syndrome (i.e., sleep disturbance, poor mood, and inattention) [44]. Our findings revealed these symptoms in the participating athletes. CES treatment was associated with a significant decrease in anger, tension, and depression in mood evaluations, and choice reaction time was increased in athletes with poor sleep quality. Although some studies have revealed that CES influences depression, anxiety, attention, and concentration [45,46], the mechanism is still unclear; however, this mechanism is presumed to be related to increases in neurotransmitters and brainwave activity [47]. Gilula et al. hypothesized that the CES electrical current passes through the hypothalamus and modulates the reticular activating system to cause these psychological effects [48]. The meta-analysis and systemic review of Shekelle et al. reported evidence-based effects of CES on depression, anxiety, and insomnia in randomized controlled trials. They reported that depression and anxiety decreased after CES treatment [49]. However, small sample sizes limit the strength of the results of the systemic review [49]. A decrease in negative mood and increase in concentration is essential for optimal athletic performance. Mood and concentration improvements from CES might help athletes with poor sleep quality to manage pre-competition stress.

Precompetition stress can be burdensome to athletes or cause psychophysiological responses that impair sports performance [50]. HRV is a commonly used psychophysiological indicator for assessing athletes’ autonomic nervous system activity and psychological reactions. The function of the autonomic nervous system is modulated by the balance of sympathetic and parasympathetic nerve activities. This balance is related to psychological responses, such as depression, anxiety, and stress [51]. HF and LF represent the activity of the parasympathetic and sympathetic nervous systems, respectively [52]. Therefore, the LF/HF ratio indicates the sympathetic and parasympathetic nervous systems [52]. We found that LF and LF/HF ratio had a positive correction in CES and placebo groups, reflecting the relationship of sympathetic nerve activity and autonomic nervous system balance. Wagenseil et al. suggested that HRV may be a sensitive marker for evaluating parasympathetic activity and valuable tools for assessing CES outcomes [53]. This study observed an increase in parasympathetic nervous system activity (i.e., an increase in HF) and a decrease in sympathetic nervous system activity (i.e., a decrease in LF) after the 2-week CES treatment. However, the opposite results occurred in the placebo group. A study indicated a significant association between negative mood and HRV [51]. Decreases in negative moods (i.e., anger, tension, and depression) were associated with autonomic nervous system activity changes after use of CES; therefore, CES can be beneficial for athletes with poor sleep quality before a competition. We also found that the change in total mood disturbance negatively correlated with sleep efficiency. Kennerly et al. indicated that CES could increase serotonin, dopamine, and norepinephrine levels in the brain and reduce cortisol levels [54]. Patients with insomnia were in a state of “alertness, but relaxation” revealed by alpha brain wave increases, and the delta brain wave decreases on an electroencephalograph [54]. These physiological responses could cause the emotional improvement, and the balance of parasympathetic and sympathetic nerve activities observed to be stable in this study.

Kirsch et al. used CES for military service people and veterans and found self-reported improvement of >25% for depression, anxiety, and insomnia [26]. Studies have also been performed to validate the CES mechanism facilitating sleep. CES could increase the production of neurotransmitters such as serotonin, dopamine, dehydroepiandrosterone, and endorphins and stabilize the nervous system [14,55]. An animal study revealed that CES could affect the hyperpolarization of postsynaptic potentials, adjust neurotransmitter levels, and increase inhibitory nerve signals [56]. However, the mechanism of CES that facilitates sleep is still unclear. Stress sources are factors that influence sleep quality and cause psychophysiological responses. In the current study, an upcoming competition was a source of progressively increasing stress for the athletes that impaired sleep efficiency. Sleep architecture and PSG findings in the CES and placebo groups were nearly normal. Although the difference in sleep efficiency between the CES group and the placebo group was nonsignificant, the slope of the sleep efficiency regression curve suggested that CES had a protective effect on the decrease in sleep efficiency throughout the study.

This study has some limitations. First, psychological conditions were not controlled in participant selection. Because each athlete’s stress response to an upcoming competition differed, psychophysiological responses also differed individually. Second, detailed changes in sleep or the long-term effects of CES on sleep efficiency and psychological responses could not be determined due to the lack of detailed daily and follow-up measurements. Investigation of the long-term effects of CES on the sleep quality and sleep efficiency of athletes experiencing competition-related stress is recommended for future studies.

## 5. Conclusions

In summary, sleep problems in athletes before competition are due to psychological stress. When athletes with poor sleep quality received 2-week CES treatment before a competition, their negative emotions decreased, and choice reaction times improved. The balance of parasympathetic and sympathetic nerve activities also tended to be stable, and a positive effect against the deterioration of sleep efficiency was observed after CES. Although the changes from pretrial to posttrial were negative emotions in POMS, choice reaction time, and sleep efficiency after receiving CES, to conclude on small effect sizes for poor sleep quality in athletes before a competition, more studies are warranted.

## Figures and Tables

**Table 1:** Demographic and baseline data of the participants.

	CES Group (*n* = 20)	Placebo Group (*n* = 20)	*p* Value
Age (years)	21.55 ± 2.26	21.05 ± 1.46	0.41
Height (cm)	171.84 ± 9.66	171.17 ± 8.42	0.91
Weight (kg)	69.70 ± 12.51	70.33 ± 13.79	0.87
BMI(kg/m^2^)	23.56 ± 2.79	23.93 ± 3.97	0.73
Body fat(%)	21.29 ± 8.44	21.81 ± 7.78	0.83
Frequency of insomnia (time/week)	2.48 ± 0.94	2.71 ± 1.81	0.56
Total PSQI	9.05 ± 2.46	9.14 ± 2.39	0.89
Sleep architecture			
REM stage (%)	20.90 ± 6.23	20.52 ± 6.75	0.85
N1 stage (%)	9.28 ± 5.37	9.77 ± 5.41	0.76
N2 stage (%)	58.77 ± 8.73	56.80 ± 10.94	0.52
N3 stage (%)	11.14 ± 9.21	12.90 ± 11.91	0.59
N4 stage (%)	0.01 ± 0.01	0.01 ± 0.01	0.98
Periodic limb movement	2.01 ± 7.93	1.26 ± 2.44	0.68
Lowest SpO_2_ (%)	91.14 ± 2.68	92.09 ± 2.11	0.21
AHI (times/h)	2.88 ± 5.75	1.01 ± 1.16	0.15
Sleep onset latency (min)	15.36 ± 20.24	17.81 ± 16.77	0.67
Sleep efficiency (%)	88.01 ± 11.56	87.20 ± 7.49	0.79
ESS	9.38 ± 3.81	9.71 ± 4.02	0.78

BMI, body mass index; PSQI, Pittsburgh Sleep Quality Index; REM, rapid eye movement; AHI, Apnea-hypopnea index; SpO_2_, Oxygen saturation; ESS, Epworth sleepiness scale.

**Table 2:** Outcomes of biochemistry analysis of the two groups.

	CES Group (*n* = 20)				Placebo Group (*n* = 20)			
	Pre-	95% CI	Post-	95% CI	Pre-	95% CI	Post-	95% CI
Blood Urea Nitrogen (mg/dL)	16.01 ± 3.91	14.29–17.72	15.01 ± 3.90	13.30–16.71	15.10 ± 3.49	13.57–16.63	15.24 ± 3.25	13.81–16.66
Creatine Phosphate (U/L)	240.19 ± 198.58	153.16–327.22	206.95 ± 193.08	122.33–291.56	239.67 ± 154.62	171.90–307.43	239.71 ± 112.38	190.458–288.96
Testosterone (ng/dL)	409.60 ± 334.39	263.050–556.15	396.39 ± 325.19	253.87–538.90	441.34 ± 340.38	292.16–590.51	452.89 ± 331.38	307.65–598.12
Cortisol (ug/dL)	12.09 ± 3.45	10.57–13.60	12.19 ± 3.79	10.52–13.85	13.59 ± 4.58	11.58–15.59	13.49 ± 4.89	11.347–15.63
Saliva pH	7.25 ± 0.36	7.09–7.40	7.09 ± 0.51	6.86–7.31	7.29 ± 0.40	7.11–7.46	7.28 ± 0.42	7.09–7.46

**Table 3:** Outcomes of POMS, HRV analysis, and reaction time test between groups.

	CES Group (*n* = 20)				Placebo Group (*n* = 20)			
	Pre-	95% CI	Post-	95% CI	Pre-	95% CI	Post-	95% CI
POMS								
Confusion	0.70 ± 0.83	0.33–1.06	0.38 ± 0.45	0.18–0.57	0.98 ± 1.18	0.46–1.49	0.74 ± 0.96	0.31–1.16
Fatigue	0.96 ± 0.84	0.59–1.32	0.73 ± 0.65	0.44–1.01	1.23 ± 0.96	0.80–1.65	1.12 ± 1.10	0.638–1.60
Anger	0.36 ± 0.45	0.16–0.55	0.11 ± 0.20 *	0.02–0.19	0.24 ± 0.45	0.04–0.43	0.26 ± 0.47	0.05–0.46
Tension	1.62 ± 0.97	1.19–2.04	1.12 ± 0.74 *	0.79–1.44	1.65 ± 1.19	1.12–2.17	1.27 ± 1.05	0.81–1.73
Depression	1.67 ± 1.06	1.20–2.13	0.81 ± 0.75 *	0.48–1.13	1.38 ± 1.40	0.76–1.99	1.14 ± 1.28	0.57–1.70
Vigor	2.20 ± 0.82	1.84–2.55	2.29 ± 0.92	1.88–2.69	1.77 ± 0.91	1.37–2.16	1.83 ± 0.97	1.40–2.25
Esteem	1.15 ± 0.48	0.94–1.36	1.14 ± 0.41	0.96–1.32	0.97 ± 0.41	0.79–1.15	1.02 ± 0.48	0.81–1.23
Total mood disturbance	92.05 ± 17.37	84.43–99.66	84.01 ± 16.99	76.56–91.45	99.14 ± 22.12	89.44–108.83	94.52 ± 21.42	85.13–103.91
HRV analysis								
Heart rate (bpm)	62.31 ± 9.58	58.11–66.50	62.08 ± 9.44	57.94–66.21	62.50 ± 9.41	58.37–66.62	61.18 ± 10.76	56.46–65.89
SDNN (ms)	65.68 ± 30.38	52.36–78.99	68.03 ± 27.08	56.16–79.89	73.44 ± 49.85	51.59–95.28	76.62 ± 29.66	63.62–89.61
LF (%)	57.11 ± 16.12	50.04–64.17	49.37 ± 18.34	41.33–57.40	54.24 ± 16.70	46.92–61.55	59.85 ± 14.41	53.53–66.16
HF (%)	42.87 ± 16.10	35.81–49.92	50.61 ± 18.35	42.56–58.65	45.57 ± 16.76	38.22–52.91	40.14 ± 14.42	33.82–46.46
LF/HF	1.80 ± 1.39	1.19–2.40	1.21 ± 0.73	0.89–1.53	1.76 ± 1.87	0.94–2.58	1.85 ± 1.15 ^#^	1.34–2.35
Reaction time test								
Simple reaction time (ms)	322.25 ± 40.78	304.37–340.12	306.95 ± 29.16	294.17–319.73	307.05 ± 29.18	294.26–319.83	307.85 ± 32.83	293.46–322.23
Choice reaction time (ms)	420.85 ± 41.22	402.78–438.91	399.90 ± 36.71 *^#^	383.81–415.98	419.35 ± 46.12	399.13–439.56	428.15 ± 48.73	406.79–449.50

* *p* < 0.05, pre- vs. post-; ^#^ *p* < 0.05, CES group vs. placebo group. HRV, heart rate variability; SDNN, the standard deviation of the normal-to-normal interval; LF, low-frequency; HF, high-frequency.

**Table 4:** Sleep parameters of the two groups as measured through Actigraphy.

	CES Group (*n* = 20)				Placebo Group (*n* = 20)			
	Pre-	95% CI	Post-	95% CI	Pre-	95% CI	Post-	95% CI
Sleep latency	2.19 ± 1.53	1.51–2.86	3.52 ± 2.94	2.23–4.80	2.57 ± 2.13	1.63–3.50	2.62 ± 2.32	1.60–3.63
Sleep efficiency (%)	87.94 ± 6.76	84.97–90.90	81.75 ± 9.62 *	77.53–85.96	89.60 ± 9.19	85.57–93.62	81.36 ± 9.64 *	77.13–85.58
Total minutes in bed (min)	370.01 ± 52.94	346.80–393.21	372.86 ± 75.06	339.96– 405.75	392.19 ± 38.29	375.40–408.97	320.62 ± 74.08 *^#^	288.15–353.08
Total sleep time (min)	327.33 ± 63.35	299.56–355.09	306.71 ± 85.02	269.44–343.97	352.29 ± 56.69	327.44–377.13	262.71 ± 77.35 *	228.81–296.61
WASO	40.48 ± 22.06	30.81–50.14	62.62 ± 35.70	46.97–78.26	37.33 ± 35.63	21.71–52.94	55.29 ± 28.34	42.87–67.71
Number of awakenings	15.03 ± 5.74	12.51–17.54	19.43 ± 10.12 *	14.99–23.86	13.29 ± 7.44	10.02–16.55	21.62 ± 6.79 *	18.64–24.59
Average awakening length	2.61 ± 1.05	2.15–3.07	3.61 ± 2.84	2.36–4.85	2.35 ± 1.36	1.75–2.94	2.54 ± 0.90	2.14–2.93
Movement index	13.18 ± 5.33	10.84–15.51	20.82 ± 17.64	13.08–28.55	15.59 ± 11.63	10.49–20.68	16.78 ± 10.40	12.22–21.33
Fragmentation index	13.03 ± 10.05	8.62–17.43	14.74 ± 8.60	10.97–18.50	11.76 ± 7.67	8.39–15.12	15.20 ± 8.37	11.53–18.86
Sleep fragmentation index	26.21 ± 13.74	20.18–32.23	35.56 ± 16.84	28.18–42.94	27.35 ± 14.76	20.88–33.81	31.98 ± 14.77	25.507–38.45

* *p* < 0.05, pre- vs. post-; ^#^ *p* < 0.05, CES group vs. placebo group; WASO, wake after sleep onset.

**Table 5:** Within-subject correlation between the related variables in CES and placebo groups.

	Total Mood Disturbance	LF (%)	HF (%)	LF/HF	Simple Reaction Time (ms)	Choice Reaction Time (ms)
CES group						
LF (%)	−0.26					
HF (%)	0.26	−1.00 *				
LF/HF	−0.13	0.95 *	−0.95 *			
Simple reaction time (ms)	0.16	0.13	−0.13	0.12		
Choice reaction time (ms)	0.28	−0.11	0.11	−0.03	0.35	
Sleep efficiency (%)	−0.51 *	0.01	−0.01	−0.01	−0.11	−0.41
Placebo group						
LF (%)	0.29					
HF (%)	−0.30	−1.00 *				
LF/HF	0.10	0.89 *	−0.89 *			
Simple reaction time (ms)	−0.08	−0.01	0.01	0.26		
Choice reaction time (ms)	0.02	−0.02	0.03	−0.01	0.26	
Sleep efficiency (%)	−0.21	−0.09	0.10	−0.12	−0.05	0.27

* *p* < 0.05; LF, low-frequency; HF, high-frequency.

## Data Availability

Data is contained within the article.

## References

[1] Wilkes J.R., Walter A.E., Chang A.M., Miller S.J., Sebastianelli W.J., Seidenberg P.H., Slobounov S. (2021). Effects of sleep disturbance on functional and physiological outcomes in collegiate athletes: A scoping review. Sleep Med..

[2] Juliff L.E., Halson S.L., Peiffer J.J. (2015). Understanding sleep disturbance in athletes prior to important competitions. J. Sci. Med. Sport.

[3] Costa J.A., Figueiredo P., Nakamura F.Y., Rebelo A., Brito J. (2021). Monitoring individual sleep and nocturnal heart rate variability indices: The impact of training and match schedule and load in high-level female soccer players. Front. Physiol..

[4] Costa J., Figueiredo P., Nakamura F., Rago V., Rebelo A., Brito J. (2019). Intra-individual variability of sleep and nocturnal cardiac autonomic activity in elite female soccer players during an international tournament. PLoS ONE.

[5] Figueiredo P., Costa J., Lastella M., Morais J., Brito J. (2021). Sleep indices and cardiac autonomic activity responses during an international tournament in a youth national soccer team. Int. J. Environ. Res. Public Health.

[6] Leeder J., Glaister M., Pizzoferro K., Dawson J., Pedlar C. (2012). Sleep duration and quality in elite athletes measured using wristwatch actigraphy. J. Sports Sci..

[7] Lastella M., Lovell G.P., Sargent C. (2014). Athletes’ precompetitive sleep behaviour and its relationship with subsequent precompetitive mood and performance. Eur. J. Sport Sci..

[8] Mamiya A., Morii I., Goto K. (2021). Effects of partial sleep deprivation after prolonged exercise on metabolic responses and exercise performance on the following day. Phys. Act. Nutr..

[9] Meeusen R., Duclos M., Foster C., Fry A., Gleeson M., Nieman D., Raglin J., Rietjens G., Steinacker J., Urhausen A. (2013). Prevention, diagnosis, and treatment of the overtraining syndrome: Joint consensus statement of the European College of Sport Science and the American College of Sports Medicine. Med. Sci. Sports Exerc..

[10] Dinges D.F., Pack F., Williams K., Gillen K.A., Powell J.W., Ott G.E., Aptowicz C., Pack A.I. (1997). Cumulative sleepiness, mood disturbance, and psychomotor vigilance performance decrements during a week of sleep restricted to 4–5 hours per night. Sleep.

[11] Jarraya M., Jarraya S., Chtourou H., Souissi N., Chamari K. (2013). The effect of partial sleep deprivation on the reaction time and the attentional capacities of the handball goalkeeper. Biol. Rhythm Res..

[12] Blumert P.A., Crum A.J., Ernsting M., Volek J.S., Hollander D.B., Haff E.E., Haff G.G. (2007). The acute effects of twenty-four hours of sleep loss on the performance of national-caliber male collegiate weightlifters. J. Strength Cond. Res..

[13] Kirsch D.L., Nichols F. (2013). Cranial electrotherapy stimulation for treatment of anxiety, depression, and insomnia. Psychiatr. Clin. N. Am..

[14] Nitsche M.A., Fricke K., Henschke U., Schlitterlau A., Liebetanz D., Lang N., Henning S., Tergau F., Paulus W. (2003). Pharmacological modulation of cortical excitability shifts induced by transcranial direct current stimulation in humans. J. Physiol..

[15] Klawansky S., Yeung A., Berkey C., Shah N., Phan H., Chalmers T.C. (1995). Meta-analysis of randomized controlled trials of cranial electrostimulation. Efficacy in treating selected psychological and physiological conditions. J. Nerv. Ment. Dis..

[16] Holubec J.T. (2009). Cumulative response from cranial electrotherapy stimulation (CES) for chronic pain. Pract. Pain Manag..

[17] Erlacher D., Ehrlenspiel F., Adegbesan O.A., El-Din H.G. (2011). Sleep habits in German athletes before important competitions or games. J. Sports Sci..

[18] Herman D., Macknight J.M., Stromwall A.E., Mistry D.J. (2011). The international athlete—Advances in management of jet lag disorder and anti-doping policy. Clin. Sports Med..

[19] Tsai P.S., Wang S.Y., Wang M.Y., Su C.T., Yang T.T., Huang C.J., Fang S.C. (2005). Psychometric evaluation of the Chinese version of the Pittsburgh Sleep Quality Index (CPSQI) in primary insomnia and control subjects. Qual. Life Res..

[20] Omobomi O., Quan S.F. (2018). A Requiem for the clinical use of the Epworth sleepiness scale. J. Clin. Sleep Med..

[21] Peng L.L., Li J.R., Sun J.J., Li W.Y., Sun Y.M., Zhang R., Yu L.L. (2011). Reliability and validity of the simplified Chinese version of Epworth sleepiness scale. Chin. J. Otorhinolaryngol. Head Neck Surg..

[22] Jafari B., Mohsenin V. (2010). Polysomnography. Clin. Chest Med..

[23] Swinbourne R., Gill N., Vaile J., Smart D. (2016). Prevalence of poor sleep quality, sleepiness and obstructive sleep apnoea risk factors in athletes. Eur. J. Sport Sci..

[24] Feighner J.P., Brown S.L., Olivier J.E. (1973). Electrosleep therapy. A controlled double blind study. J. Nerv. Ment. Dis..

[25] Erdfelder E., Faul S., Buchner A. (1996). G-power: A general power analysis program. Behav. Res. Methods Instrum. Comp..

[26] Kirsch D.L., Price L.R., Nichols F., Marksberry J.A., Platoni K.T. (2014). Military service member and veteran self reports of efficacy of cranial electrotherapy stimulation for anxiety, posttraumatic stress disorder, insomnia, and depression. US Army Med. Dep. J..

[27] Perroni F., Migliaccio S., Borrione P., Vetrano M., Amatori S., Sisti D., Rocchi M.B.L., Salerno G., Vescovo R.D., Cavarretta E. (2020). Can haematological and hormonal biomarkers predict fitness parameters in youth soccer players? A pilot study. Int. J. Environ. Res. Public Health.

[28] Brancher J.A., Morodome F., Madalena I.R., Reis C.L.B., Von Held R., Antunes L.A.A., Winckler C., Salgueirosa F., Neto Z.C.O., Storrer C.L.M. (2021). Salivary pH and oral health of Brazilian para-athletes: Saliva and oral health of para-athletes. Spec. Care Dentist..

[29] Stoet G. (2010). PsyToolkit: A software package for programming psychological experiments using Linux. Behav. Res. Methods.

[30] Kim J., Gabriel U., Gygax P. (2019). Testing the effectiveness of the Internet-based instrument PsyToolkit: A comparison between web-based (PsyToolkit) and lab-based (E-Prime 3.0) measurements of response choice and response time in a complex psycholinguistic task. PLoS ONE.

[31] Chen K.M., Snyder M., Krichbaum K. (2002). Translation and equivalence: The Profile of Mood States Short Form in English and Chinese. Int. J. Nurs. Stud..

[32] McNair D.M., Lorr M., Droppleman L.F. (1992). EdITS Manual of the Profile of Mood States.

[33] Tsutsui Y., Mizuno J., Sunada K. (2018). Does the aroma of a patient’s preferred dental topical anaesthetic affect anxiety, fear, and autonomic nervous system activity prior to dental local anaesthesia? A randomized trial. Flavour Fragr. J..

[34] Beauchaine T.P., Thayer J.F. (2015). Heart rate variability as a transdiagnostic biomarker of psychopathology. Int. J. Psychophysiol..

[35] Degroote L., Hamerlinck G., Poels K., Maher C., Crombez G., De Bourdeaudhuij I., Vandendriessche A., Curtis R.G., DeSmet A. (2020). Low-cost consumer-based trackers to measure physical activity and sleep duration among adults in free-living conditions: Validation study. JMIR Mhealth Uhealth.

[36] Sargent C., Lastella M., Halson S.L., Roach G.D. (2016). The validity of activitymonitors for measuring sleep in elite athletes. J. Sci. Med. Sport.

[37] Sadeh A., Sharkey K.M., Carskadon M.A. (1994). Activity-based sleep-wake identification: An empirical test of methodological issues. Sleep.

[38] Cohen J. (1992). A power primer. Psychol. Bull..

[39] Hopkins W.G., Marshall S.W., Batterham A.M., Hanin J. (2009). Progressive statistics for studies in sports medicine and exercise science. Med. Sci. Sports Exerc..

[40] Rose K.M., Taylor A.G., Bourguignon C., Utz S.W., Goehler L.E. (2008). Cranial electrical stimulation: Potential use in reducing sleep and mood disturbances in persons with dementia and their family caregivers. Fam. Community Health.

[41] Anderson T., Lane A.R., Hackney A.C. (2016). Cortisol and testosterone dynamics following exhaustive endurance exercise. Eur. J. Appl. Physiol..

[42] Martin T.G., Pata R.W., D’Addario J., Yuknis L., Kingston R., Feinn R. (2015). Impact of age on haematological markers pre- and post-marathon running. J. Sports Sci..

[43] Nunes J.A., Moreira A., Crewther B.T., Nosaka K., Viveiros L., Aoki M.S. (2014). Monitoring training load, recovery-stress state, immune-endocrine responses, and physical performance in elite female basketball players during a periodized training program. J. Strength Cond. Res..

[44] Halson S.L. (2014). Monitoring training load to understand fatigue in athletes. Sports Med..

[45] Barclay T.H., Barclay R.D. (2014). A clinical trial of cranial electrotherapy stimulation for anxiety and comorbid depression. J. Affect. Disord..

[46] Southworth S. (1999). A study of the effects of cranial electrical stimulation on attention and concentration. Integr. Physiol. Behav. Sci..

[47] Zaghi S., Acar M., Hultgren B., Boggio P.S., Fregni F. (2010). Non-invasive brain stimulation with low-intensity electrical currents: Putative mechanisms of action for direct and alternating current stimulation. Neuroscientist.

[48] Gilula M.F., Kirsch D.L. (2005). Cranial electrotherapy stimulation review: A safer alternative to psychopharmaceuticals in the treatment of depression. J. Neurother..

[49] Shekelle P.G., Cook I.A., Miake-Lye I.M., Booth M.S., Beroes J.M., Mak S. (2018). Benefits and harms of cranial electrical stimulation for chronic painful conditions, depression, anxiety, and insomnia: A systematic review. Ann. Intern. Med..

[50] Wang H.T., Tai H.L., Yang C.C., Chen Y.S. (2020). Acute effects of self-selected music intervention on golf performance and anxiety level in collegiate golfers: A crossover study. Int. J. Environ. Res. Public Health.

[51] Fortes L.S., da Costa B.D., Paes P.P., do Nascimento Júnior J.R., Fiorese L., Ferreira M.E. (2017). Influence of competitiveanxiety on heart rate variability in swimmers. J. Sports Sci. Med..

[52] Pinna G.D., Maestri R., Torunski A., Danilowicz-Szymanowicz L., Szwoch M., La Rovere M.T., Raczak G. (2007). Heart rate variability measures: A fresh look at reliability. Clin. Sci..

[53] Wagenseil B., Garcia C., Suvorov A.V., Fietze I., Penzel T. (2018). The effect of cranial electrotherapy stimulation on sleep in healthy women. Physiol. Meas..

[54] Kennerly R.C. (2004). QEEG analysis of cranial electrotherapy: A pilot study. J. Neurother..

[55] Yennurajalingam S., Kang D.H., Hwu W.J., Padhye N.S., Masino C., Dibaj S.S., Liu D.D., Williams J.L., Lu Z., Bruera E. (2018). Cranial electrotherapy stimulation for the management of depression, anxiety, sleep disturbance, and pain in patients with advanced cancer: A preliminary study. J. Pain Symptom Manag..

[56] Pozos R.S., Richardson A.W., Kaplan H.M. (1969). Mode of production and locus of action of electroanesthesia in dogs. Anesth. Analg..

